# Bellwethers of change: population modelling of North Pacific humpback whales from 2002 through 2021 reveals shift from recovery to climate response

**DOI:** 10.1098/rsos.231462

**Published:** 2024-02-28

**Authors:** Ted Cheeseman, Jay Barlow, Jo Marie Acebes, Katherina Audley, Lars Bejder, Caitlin Birdsall, Olga Solis Bracamontes, Amanda L. Bradford, Josie Byington, John Calambokidis, Rachel Cartwright, Jen Cedarleaf, Andrea Jacqueline García Chavez, Jens Currie, Rouenne Camille De Castro, Joëlle De Weerdt, Nicole Doe, Thomas Doniol-Valcroze, Karina Dracott, Olga Filatova, Rachel Finn, Kiirsten R. Flynn, John Ford, Astrid Frisch-Jordán, Chris Gabriele, Beth Goodwin, Craig Hayslip, Jackie Hildering, Marie C. Hill, Jeff K. Jacobsen, M. Esther Jiménez-López, Meagan Jones, Nozomi Kobayashi, Marc Lammers, Edward Lyman, Mark Malleson, Evgeny Mamaev, Pamela Martínez Loustalot, Annie Masterman, Craig O. Matkin, Christie McMillan, Jeff Moore, John Moran, Janet L. Neilson, Hayley Newell, Haruna Okabe, Marilia Olio, Christian D. Ortega-Ortiz, Adam A. Pack, Daniel M. Palacios, Heidi Pearson, Ester Quintana-Rizzo, Raul Ramírez Barragán, Nicola Ransome, Hiram Rosales-Nanduca, Fred Sharpe, Tasli Shaw, Ken Southerland, Stephanie Stack, Iain Staniland, Janice Straley, Andrew Szabo, Suzie Teerlink, Olga Titova, Jorge Urban-Ramirez, Martin van Aswegen, Marcel Vinicius, Olga von Ziegesar, Briana Witteveen, Janie Wray, Kymberly Yano, Igor Yegin, Denny Zwiefelhofer, Phil Clapham

**Affiliations:** ^1^ Marine Ecology Research Centre, Southern Cross University, Lismore, New South Wales, Australia; ^2^ Happywhale, Santa Cruz, CA, USA; ^3^ Marine Mammal Institute, Oregon State University, Newport, OR, USA; ^4^ BALYENA.ORG, Brgy. Pangdan, Jagna, Bohol, The Philippines; ^5^ Whales of Guerrero, Portland, OR, USA; ^6^ Marine Mammal Research Program, Hawai'i Institute of Marine Biology, University of Hawai'i at Manoa, Kaneohe, HI, USA; ^7^ Marine Education and Research Society, Port McNeill, British Columbia, Canada; ^8^ University of Guadalajara, Guadalajara, Jalisco, Mexico; ^9^ NOAA Fisheries Pacific Islands Fisheries Science Center, Honolulu, HI, USA; ^10^ Pacific Wildlife Foundation Canada, Port Moody, British Columbia, Canada; ^11^ Cascadia Research Collective, Olympia, WA, USA; ^12^ The Keiki Kohola Project, Delray Beach, FL, USA; ^13^ California State University Channel Islands, Camarillo, CA, USA; ^14^ University of Alaska Southeast, Sitka Campus, Sitka, AK, USA; ^15^ Pacific Whale Foundation, Wailuku, HI, USA; ^16^ Association ELI-S, Gujan-Mestras, France; ^17^ Vrije Universiteit, Brussels, Belgium; ^18^ Pacific Biological Station, Fisheries and Oceans Canada, Nanaimo, British Columbia, Canada; ^19^ Ocean Wise, Vancouver, British Columbia, Canada; ^20^ Department of Biology, University of Southern Denmark, Odense, Denmark; ^21^ Hawaiian Islands Humpback Whale National Marine Sanctuary, Kīhei, HI, USA; ^22^ Ecología y Conservación de Ballenas, AC, Puerto Vallarta, Jalisco, Mexico; ^23^ Hawai'i Marine Mammal Consortium, Waimea, HI, USA; ^24^ Glacier Bay National Park and Preserve, Gustavus, AK, USA; ^25^ Eye of the Whale Marine Mammal Research, Kamuela, HI, USA; ^26^ Cooperative Institute for Marine and Atmospheric Research, Research Corporation of the University of Hawai'i, Honolulu, HI, USA; ^27^ VE Enterprises, McKinleyville, CA, USA; ^28^ Departamento Académico de Ingeniería en Pesquerías, Universidad Autónoma de Baja California Sur, La Paz, BCS, Mexico; ^29^ Whale Trust, Puunene, HI, USA; ^30^ Okinawa Churashima Foundation, Okinawa, Japan; ^31^ Center for Whale Research, Friday Harbour, WA, USA; ^32^ FGBU Gosudarstvennyj zapovednik Komandorskij, Commander Islands, Kamchatka Krai, Russia; ^33^ Universidad Autonoma de Baja California Sur, La Paz, BCS, Mexico; ^34^ National Marine Fisheries Service, NOAA, Auke Bay Laboratories, Alaska Fisheries Science Center, Juneau, AK, USA; ^35^ North Gulf Oceanic Society, Homer, AK, USA; ^36^ NOAA Fisheries Southwest Fisheries Science Center, La Jolla, CA, USA; ^37^ Faculty of Marine Sciences, Universidad de Colima, Colima, Mexico; ^38^ Department of Psychology, University of Hawai'i at Hilo, Hilo, HI, USA; ^39^ The Dolphin Institute, Hilo, HI, USA; ^40^ University of Alaska Southeast, Juneau, AK, USA; ^41^ Emmanuel College, Boston, MA, USA; ^42^ College of Sustainable Aquatic Ecosystems, Harry Butler Institute, Murdoch University, Western Australia, Australia; ^43^ McCowan Lab, University of California Davis, Davis, CA, USA; ^44^ Humpback Whales of the Salish Sea, Duncan, British Columbia, Canada; ^45^ International Whaling Commission, Impington, UK; ^46^ Alaska Whale Foundation, Petersburg, AK, USA; ^47^ Juneau Flukes, Juneau, AK, USA; ^48^ NOAA Fisheries Alaska Regional Office, Juneau, AK, USA; ^49^ A. N. Severtsov Institute of Ecology and Evolution of the Russian Academy of Sciences, Moscow, Russia; ^50^ Winged Whale Research, Homer, AK, USA; ^51^ University of Alaska Fairbanks College of Fisheries and Ocean Sciences, Fairbanks, AK, USA; ^52^ North Coast Cetacean Society, Alert Bay, British Columbia, Canada; ^53^ University of Stirling, Stirling, UK; ^54^ Seastar Scientific, Vashon, WA, USA

**Keywords:** carrying capacity, marine heatwave, mark–recapture modelling, abundance estimation, climate change, environmental variables

## Abstract

For the 40 years after the end of commercial whaling in 1976, humpback whale populations in the North Pacific Ocean exhibited a prolonged period of recovery. Using mark–recapture methods on the largest individual photo-identification dataset ever assembled for a cetacean, we estimated annual ocean-basin-wide abundance for the species from 2002 through 2021. Trends in annual estimates describe strong post-whaling era population recovery from 16 875 (± 5955) in 2002 to a peak abundance estimate of 33 488 (± 4455) in 2012. An apparent 20% decline from 2012 to 2021, 33 488 (± 4455) to 26 662 (± 4192), suggests the population abruptly reached carrying capacity due to loss of prey resources. This was particularly evident for humpback whales wintering in Hawai‘i, where, by 2021, estimated abundance had declined by 34% from a peak in 2013, down to abundance levels previously seen in 2006, and contrasted to an absence of decline in Mainland Mexico breeding humpbacks. The strongest marine heatwave recorded globally to date during the 2014–2016 period appeared to have altered the course of species recovery, with enduring effects. Extending this time series will allow humpback whales to serve as an indicator species for the ecosystem in the face of a changing climate.

## 1. Introduction

Population monitoring has become increasingly important in conservation biology as anthropogenic activities and climate change continue to impact marine ecosystems [1–5]. Abundance trends provide critical insights into the dynamics of animal populations, enabling a better understanding of the ecological interactions and underlying drivers that influence their distribution, abundance and life history [6,7]. Abundance estimation is the basis for assessing the current protection status for humpback whales in the USA [8] and Canada [9] and has helped the understanding of anthropogenic effects such as ship strikes on blue whales [10]. For marine mammal populations exploited to near extinction, abundance estimation has served as an indispensable tool for decision-makers, supporting the development of effective conservation and management strategies aimed at protecting these animals and the ecosystems they inhabit [11,12]. Understanding population trends is essential for addressing the growing challenges faced by marine mammals in a rapidly changing world [13]; however, detecting population changes can be difficult due to the imprecision of most marine mammal abundance estimates and the infrequent intervals at which most populations are surveyed [14]. For large cetaceans, such as baleen whales, these issues are largely due to the costs and logistical challenges associated with surveying vast, often remote marine environments, as well as elusive and often cryptic animal behaviour [15]. Limited available data may result in poor model parameter estimation and reduced predictive power, ultimately hindering effective conservation and management efforts [16]. Furthermore, the infrequent generation of updated population estimates may fail to capture rapid changes in whale populations due to anthropogenic disturbances or environmental fluctuations [17]. To address these challenges, we have drawn on an innovative ocean-basin-wide collaboration [18] to cost-effectively generate a time series of relatively precise abundance estimates for a long-distance migrating species, the humpback whale (*Megaptera novaeangliae*) in the North Pacific Ocean.

In the North Pacific, humpback whales are known to breed in waters off Japan, The Philippines, the Mariana Islands, Hawai‘i, Mexico and Central America [19–22]. These whales migrate to feed in coastal to continental shelf waters off Russia, the Bering Sea, Alaska, British Columbia, Washington, Oregon and California [20]. As with all large cetaceans worldwide, humpback whales in the North Pacific were the target of extensive commercial whaling until late in the twentieth century, with an estimated 31 785 whales taken from 1900 to 1976 [23,24]. Humpback whale populations in the North Pacific were severely depleted [24], with the remnant abundance crudely estimated at a low of approximately 1200–1600 individuals [25,26] around the end of humpback whale commercial catches in 1976 [24]. The end of commercial whaling created a possibility of full recovery to pre-whaling abundance; it is, however, difficult to define what full recovery means without precise estimates of pre-whaling abundance. Rice [27] suggested that the humpback population was ‘stable at about 15 000 [animals]' prior to 1905, and ‘was reduced to about 1000' by 1965. However, he gave no supporting evidence for the former figure, and the latter was estimated using catch statistics that were available at the time (either published or provided by the USSR); it is now known that the Soviet statistics were significantly under-reported [28]. As Rice stated, no one at the time had attempted to estimate the pristine population size prior to modern whaling, and the number of animals killed in various aboriginal operations prior to the twentieth century is largely unknown. Consequently, there is currently no reliable estimate of pre-whaling abundance, although calculating one is a primary aim of an ongoing Comprehensive Assessment by the Scientific Committee of the International Whaling Commission [29].

To understand population status, two previous abundance estimation studies for North Pacific humpback whales were undertaken using mark–recapture (also known as capture–recapture) techniques with synoptic datasets [30,31]. These studies depended on visual matching of photographs by skilled technicians to determine marks and recaptures of individual humpback whales based on the unique shape and pigmentation of the ventral surface of the tail (fluke) [32,33]. The first photographic mark–recapture abundance estimate for humpback whales in the full North Pacific Ocean was based on a historical database of 3650 fluke images gathered from 1990 to 1993 [34]. Important western Pacific Ocean feeding areas of Russia, the Aleutian Islands and the Bering Sea were not surveyed during the study, and because it was retrospective, sampling did not follow a systematic design. Using a geographically stratified Darroch method, that study estimated an abundance of approximately 6000 (s.e. = 474) humpback whales in 1992 from three major wintering areas (Mexico, Hawai‘i and Japan), but the authors' appraisal of likely biases suggested an adjusted estimate of approximately 8000 [34]. Assuming the same coefficient of variation (CV) as the uncorrected estimate, a minimum estimate of the standard error for the bias-corrected estimate is 632. Petersen estimates comparing wintering and summer areas suggest that this may have been an underestimate, but a more accurate Petersen model approach could not be applied uniformly due to the missing data from the unsampled summer areas.

Some of the geographical biases in the 1990–1993 sampling were corrected in the 2004–2006 project Structure of Populations, Levels of Abundance, and Status of Humpback whales (SPLASH) [20], when coordinated efforts by over 40 different research groups across the North Pacific obtained fluke identification photographs from all known wintering and summer areas in the North Pacific during three winters and two summers. The experimental design sought to apportion sampling effort in proportion to whale density to representatively sample whales in all areas. The SPLASH project's North Pacific abundance estimate was based on a Chapman–Petersen approach that compared all wintering fluke photographs with all summer fluke photographs [30]. In that study, simulations were used to correct biases associated with births and deaths (a common violation of the closed population assumption), not sampling calves and missed matches. Many of these biases were found to be offsetting, and the net estimate of resulting bias was only +3.5%. The bias-corrected SPLASH abundance estimate was 21 063 (CV = 0.04) humpback whales in 2005.

Based on evidence of ongoing recovery indicated by the SPLASH study and other studies, the species was partially delisted from the United States Endangered Species Act (ESA) in 2016 [8], and was reassessed from Threatened to Special Concern by the Committee on the Status of Endangered Wildlife in Canada in 2011 [35]. However, multiple current anthropogenic stressors including ship strikes [36], entanglements [37] and climate change [5,38–40] may limit or reverse full recovery. Our 2001–2022 study period included seasons before, during and after periods of a severe marine heatwave [41], with demonstrated effects on regional humpback whale populations [39], and signs of compounding climate change and habitat use conflict such as fisheries interactions leading to exacerbation of these existing threats [42,43].

Our current study builds on past photo-identification (photo-ID) efforts mentioned above, incorporating the historical data gathered in those studies, combined with long-term region-specific photo-ID efforts, and making broader use of opportunistic sources, especially whale watch voyage naturalists. The sheer scale of data now available is due to advances in automated image recognition fluke photo-ID matching [44], data management and research collaboration [18]. These advances have shifted the abundance estimation paradigm from data scarcity and periodic study to continuous and accessible tracking of the ocean-basin-wide population through time. Given that no single research group's data archive or effort could scale and maintain what would be necessary for such an ongoing study, we combined research-based and community science data in a broad collaboration facilitated by the data management Web platform Happywhale.com [18]. Where data deficiency previously imposed limits on understanding, this study used a near-real-time population modelling infrastructure and with this, we aimed to evaluate population change with a robust time series of 20 annual abundance estimates from 2002 to 2021. We used the same Chapman–Petersen mark–recapture approach for abundance estimation as was used for the 2004–2006 SPLASH study [30], based on a comparison of three wintering samples with two summer samples for each calculated annual estimation. To address geographically biased sampling in pre- and post-SPLASH years, we used a sub-sampling scheme of the SPLASH years to obtain a bias correction. We examined whether the results of this study might be biased by long-term changes in migratory patterns and in the relatively large proportions of the entire population that winter in Mainland Mexico and Hawai‘i, respectively. Finally, we discussed findings of a shift in population trajectories from recovery from industrial whaling to responding to current ecosystem conditions.

## 2. Methods

### 2.1. Sample collection and matching methods

For this study, all available North Pacific humpback whale photo-ID data from a research collaboration of 46 organizations and 4292 community science contributors were aggregated within a study period of 2001 through 2022, and reconciled in a single dataset through the research collaboration and community science Web platform Happywhale.com. This dataset is fully described by Cheeseman *et al*. [18]. Photo-ID data for the purpose of this study consisted of encounters of individually identified whales, with associated dates and geographical locations. Multiple encounters of the same individual within the same season (summer or winter of a given year) were condensed to one capture occasion (capture), using the observation date closest to the midpoint of the respective season.

### 2.2. Geographical stratification

**Figure 1.**
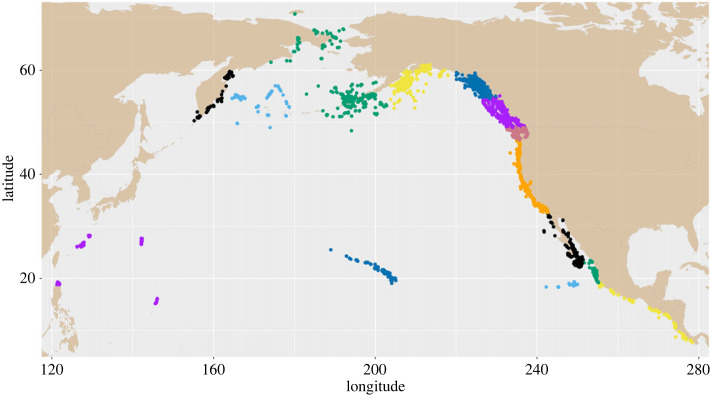
Locations of photo-identification samples from humpback whales in the North Pacific 2001–2021 colour coded by geographical strata. Summer feeding area strata north of 32°N, west to east are: Kamchatka Peninsula, Russia (black), west Bering Sea (cyan), north and western Bering Sea (green), Gulf of Alaska (yellow), southeast Alaska and northern British Columbia, Canada (blue and purple), southern British Columbia, Canada and Washington, United States (salmon), and California and Oregon, United States (orange). Wintering area strata south of 32°N, west to east are West Pacific (purple), Hawai‘i, United States (blue), Baja California, Mexico (black), Revillagigedo Islands, Mexico (cyan), Mainland Mexico (green), southern Mexico and Central America (yellow).

Each capture was assigned to a geographical stratum based on location, to segregate samples between summer (feeding) areas and winter (breeding) areas and as a method to allocate samples for a bias correction estimation described below. Geographical strata include seven summer areas and six wintering areas (tables 1 and 2; figure 1). Stratification generally followed that used in the SPLASH study [20], with strata based on similarities in migratory destinations. For example, the California and Oregon stratum was separated from the Washington and southern British Columbia stratum because whales from the former migrate almost exclusively to the coasts of Mexico and Central America, whereas a portion of the latter also migrate to the Mexican offshore islands and Hawaiian Island regions. Our large sample size allowed us to discern some migration patterns that were not apparent in the SPLASH study, which resulted in the following changes in the stratification scheme: humpback whales in southern coastal Mexico had similar distribution and migratory destinations to whales in Central America [45,46], so we separated southern Mexico from the rest of Mainland Mexico and pooled it with Central America. The ‘Russia' stratum in the SPLASH study included whales from the Kamchatka Peninsula, the Commander Islands and the Gulf of Anadyr. Based on winter migratory destinations, we revised the stratification of Russia as follows: we included the Kamchatka Peninsula as a separate stratum (Kamchatka whales migrated almost entirely to islands in the western Pacific), we combined the Commander Islands with the other western Aleutian Islands to be a new western Bering Sea stratum, we combined northern Russia coastal whales with a stratum that includes the northern and eastern Bering Sea, and samples from the Mariana Islands (which was not sampled in the SPLASH study) were pooled with the western Pacific stratum. Whale distributions in the North Pacific are continuous, with blurred boundaries across most regions (e.g. [47]), with regional delineation used here only to illustrate varied sampling over time and to develop bias-correction factors that address non-random sampling, as described below. We do not attempt to estimate abundance for each stratum and our primary abundance estimation method depends on all summer (feeding) areas pooled, and all winter (presumed breeding) areas pooled. Consequently, these geographical stratification changes have minimal impact on actual abundance estimates, serving only to align sample distributions between SPLASH study years and non-SPLASH study years. See electronic supplementary material, I, for stratum boundaries.

**Table 1.** Sample size of unique photo-identifications of humpback whale identifications in each of seven summer regions for each season-year. Regions, west to east, include the Kamchatka Peninsula (Kamchatka), the western Bering Sea (WBerSea), the eastern and northern Bering Sea and southern Chukchi Sea (E&NBerSea), the Gulf of Alaska (GulfOfAK), southeast Alaska and northern British Columbia (SEAK&NorthBC), southern British Columbia and Washington (SouthBC&WA), and California and Oregon (CA&OR).

season-year	Kamchatka	WBerSea	E&NBerSea	GulfOfAK	SEAK&NorthBC	SouthBC&WA	CA&OR
2001	0	0	85	155	78	42	240
2002	2	0	41	203	58	47	298
2003	0	1	10	147	233	23	355
2004	32	34	524	771	1305	149	302
2005	35	12	479	497	847	236	369
2006	0	9	0	147	706	148	208
2007	2	7	153	428	658	315	178
2008	12	42	195	193	615	201	296
2009	45	195	71	98	820	235	462
2010	5	679	281	216	737	164	508
2011	0	336	82	301	731	195	381
2012	5	341	181	357	572	279	513
2013	8	372	68	195	733	226	567
2014	6	156	0	208	927	134	585
2015	72	221	16	235	1032	197	653
2016	16	182	45	112	991	422	1268
2017	2	46	201	157	727	485	1306
2018	20	82	112	74	695	654	1580
2019	10	15	103	122	1471	707	1252
2020	9	3	5	68	1086	526	1120
2021	9	125	66	88	1183	535	1164

**Table 2.** Sample size of unique photo-identifications of humpback whale identifications in each of six winter regions for each season-year. Regions, west to east, include the western Pacific (WPac), the Hawaiian Islands (Hawai‘i), Baja California, Mexico (MexBajaCal), the Revillagigedo Archipelago, Mexico (MexIsl), Mainland Mexico (MexMld) and Central America and southern Mexico (CenAm&SMex).

season-year	WPac	Hawai‘i	MexBajaCal	MexIsl	MexMld	CenAm&SMex
2001	109	608	0	4	195	16
2002	130	350	0	0	221	7
2003	114	258	2	13	172	7
2004	276	1021	197	346	313	20
2005	316	1084	197	247	411	61
2006	397	1293	103	281	464	63
2007	314	1190	129	0	270	8
2008	257	886	61	0	314	54
2009	364	238	76	0	286	32
2010	316	570	100	0	312	44
2011	452	323	103	0	345	97
2012	411	269	114	0	352	50
2013	363	659	290	2	703	45
2014	454	510	442	0	607	68
2015	549	908	529	1	391	88
2016	438	430	248	0	165	60
2017	446	756	818	15	599	258
2018	414	1028	1042	0	566	218
2019	347	1628	1040	19	584	142
2020	346	1688	1317	47	583	123
2021	17	1798	638	44	719	320

### 2.3. Absolute abundance estimation methods: full North Pacific Ocean

Prior to estimating abundance, whale identification data from Happywhale were pre-processed with a custom R script [48]. For each capture, sample season was assigned as either summer or winter. As with the SPLASH study, midpoints for each capture period were defined as 1 March (for winter) and 1 August (for summer), hereafter ‘season-year'. In the rare cases of an individual encountered in multiple geographical strata in a given season-year, the individual was assigned to only one geographical stratum, based on the closest capture occasion to the seasonal midpoint.

To be comparable to the 2004–2006 SPLASH abundance estimates [30], we used the Chapman bias-corrected version of Petersen estimator [49] with wintering area samples (pooled over three consecutive years) as one capture occasion and with summer area samples (pooled over the two summers between the three years of winter samples) as the second capture occasion. Modelling has shown that this estimator is robust to sample bias created by heterogeneity in capture probabilities if the factors affecting differential availability are different in the two capture occasions [30]. We relied on the different geographical distribution and behaviour of whales between summer and winter to reduce sample bias between mark and recapture samples. Recaptures included those individuals photographed in both the pooled winter and pooled summer samples, i.e. during both ‘capture occasions'. We repeated this estimation process for 20 overlapping 3-year periods, 2002 through 2021 with data from 2001 to 2022, to produce a time series of 20 annual abundance estimates. Abundance estimates are labelled based on the year of the first summer in each 3-year period.

Although the simple abundance estimation approach outlined above should work well if the geographical areas were as representatively sampled throughout the time series as during the 2004–2006 SPLASH study, the achieved geographical distribution of samples was uneven in many years (table 1). In particular, the Revillagigedo Archipelago (MxIsl), Baja California, Mexico (MxBaja), Kamchatka, Russia (Kamch) and the eastern and western Bering Sea (BerE and BerW) were intermittently and generally under-sampled. This was expected to bias the abundance estimates outside of the SPLASH years. We therefore used a novel geographical bias correction approach, constructed as follows.

We assumed that the bias-corrected SPLASH abundance estimate of 21 063 whales [30] was an unbiased estimate for study years (2004–2006). We denoted this reference estimate as N_SPLASH_. Let the photo-ID data underpinning N_SPLASH_ be denoted Y_SPLASH_. Let *N_t_* denote the time series (2002–2021) of abundance values estimated in our analysis and let Y*_t_* be the associated photo-ID data. Note, each Y*_t_* value represented 3 years of data centred on year *t* (e.g. Y_2010_ are the data from 2009, 2010, 2011). For each *t*, we subsampled Y_SPLASH_ (call this subsample Y_SPLASH, sub(*t*)_) so that it had the same geographical distribution as Y*_t_* (see next paragraph). Y_SPLASH, sub(*t*)_ was then used to obtain an alternative SPLASH abundance estimate (N_SPLASH, alt(*t*)_). We then calculated the ratios between N_SPLASH_ and each N_SPLASH, alt(*t*)_. These ratios (*F_t_* = N_SPLASH_/N_SPLASH, alt(*t*)_) were interpreted as the amount of bias associated with the geographically biased subsamples and were applied as correction factors to the uncorrected annual Chapman estimates ($N_{t}^{\prime }$), to obtain corrected estimates, i.e. $N_{t}=N_{t}^{\prime }\times F_{t}$.

To generate subsamples from the SPLASH data (Y_SPLASH, sub(*t*)_) with the same geographical distributions as the Y*_t_*, we used a largest common sample size (LCSS) approach. We calculated the number of unique whale identifications in all summer and wintering geographical strata for both the SPLASH period (2004–2006) and for the estimation period (*t* − 1 to *t* + 1). For each summer and wintering stratum we then calculated the largest sample size that could be taken from both Y_SPLASH_ and Y*_t_*. We then subsampled both datasets by randomly sampling this LCSS from each (without replacement) to obtain Y_SPLASH, sub(*t*)_ and Y*_t_*_(sub)_. The Chapman–Petersen estimator was used with these data subsets to obtain N_SPLASH, alt(*t*)_ and $N_{t}^{\prime }$, which were used to find *F_t_* and *N_t_* (see previous paragraph).

The primary assumptions of this bias-correction approach are that (i) the SPLASH abundance estimate itself is not biased by non-representative geographical sampling, (ii) migration patterns have not changed since the SPLASH period, and (iii) the geographical distribution of whales has not changed during the course of the time series, i.e. that they have remained proportionally distributed throughout the study area as they were during the SPLASH period. The assumption of geographical sampling bias in the SPLASH study was addressed as part of that study [20,30]. The second assumption, of migration pattern consistency, was evaluated by comparing migratory destinations of southeast Alaska and northern British Columbia from two samples from different time periods, 2004–2006 and 2019–2021, three seasons each. The third assumption, of consistency of geographical distribution of whales, was evaluated through an ANOVA test of Hawai‘i versus Mainland Mexico annual mean relative abundance estimates over the study period to detect shifts in differential population growth rates between the two most sampled wintering areas.

### 2.4. Relative abundance estimation methods: Hawai‘i and Mexico

To add resolution to understanding humpback whale abundance over the study period, we estimated a time series of abundances for two wintering areas which were relatively well sampled in most years (Hawai‘i and Mainland Mexico, table 2) with consistent research effort over the study period, and which comprised a high proportion of total humpback whale abundance in the North Pacific, with migratory contributions from multiple feeding areas. Chapman–Petersen abundance estimates were made for both wintering areas based on photo-ID samples in consistently well-sampled feeding areas. Feeding areas were selected based on (i) being a substantial migratory contribution to the given wintering area [20] and (ii) having a consistently high sample size of identified whales. The Hawai‘i estimate was based on using that wintering area as one sampling occasion and the southeast Alaska/northern British Columbia feeding area stratum as the second sampling occasion. The Mainland Mexico estimate was based on using that wintering area as one sampling occasion and the California/Oregon feeding area stratum as the second sampling occasion. Humpback whales in both of these paired wintering and feeding areas exhibited elements of migratory herd units [50] with wintering areas shared with whales from multiple feeding areas, and feeding areas populated with whales almost entirely from their respective paired wintering areas. As with the ocean-basin-wide model, estimates were based on running 3-year periods with two feeding area samples and three wintering area samples. To interpret these abundance estimates as absolute abundance would have required that the wintering area sample was a random sample of the migratory whales from all feeding areas that contributed to it. Because that assumption was not evaluated in the scope of this study, we treated our estimates as an index of relative abundance.

### 2.5. Standard error estimation

To estimate standard errors (s.e.) in abundance estimates, we used a jackknife approach [51]. For both the bias-corrected estimates for the entire North Pacific and for the estimates of relative abundance for the Hawaiian Island and Mainland Mexico strata, jackknife samples were taken by randomly excluding 5% of the fluke identifications (without replacement, after season-year duplicates were excluded for each geographical stratum). The estimation of abundance (including the bias correction) was repeated for each of the 20 jackknife samples, and s.e. were estimated using the standard formula:

$$s.e.(\hat{x})=\;\sqrt{\frac{n-1}{n}\;\;\overset{n}{\underset{i=1}{\sum }}\;{\left(\hat{x_{i}}-\;{\bar{x}}_{\mathrm{jack}}\right)}^{2}},$$

where *n* was the number of jackknife estimates, $\hat{x_{i}}$ was the *i*th jackknife estimate, and ${\bar{x}}_{\mathrm{jack}}$ was the mean of the jackknife samples. The 2004–2006 SPLASH abundance estimate was used as a reference for correcting the bias for all other sample periods. To include the uncertainty in the SPLASH bias correction estimate (CV = 0.04 [30]), the CV of the bias-corrected abundance was estimated as the square root of the sum of the squared CV of the uncorrected abundance estimate plus the squared CV of the SPLASH estimate.

## 3. Results

### 3.1. Sampling distribution

The full 2001–2022 dataset consisted of 30 484 individual humpback whales in 132 684 unique season-year encounters, distilled from 192 869 unique encounters. The number of unique individuals that were photographically sampled each season-year varied from 1940 to 5668, and from 1252 to 3236 for the 21 winters and summers, respectively (table 3). Photo-ID samples were post-stratified into seven feeding areas (table 1) and six wintering areas (table 2). Sample sizes varied among seasons and regions due to varying effort; 20 of 294 season-regions had no effort and zero identifications. Kamchatka and the Mexican Islands regions were consistently under-sampled, with an average of 14 and 49 seasonal identifications, respectively. Hawai‘i (833), California/Oregon (648) and southeast Alaska/northern British Columbia (772) averaged the highest seasonal identifications, resulting from both high abundance and high effort. Across all seasons and regions, we found a mean of 448 unique identifications per season, and a maximum of 1798 unique identifications in one season. Sample sizes increased on average fourfold over the years of the study period, with the highest number of unique identifications from 2017 onward (table 3). Further description of this sample distribution is found in [18].

**Table 3.** Sample size of unique summer and winter area whales (three and two seasons pooled, respectively), number of between-season matches, and resulting North Pacific abundance estimates without and with bias corrections for non-representative geographical sampling. Standard errors are estimated from jackknife samples (*n* = 20). Between season match rates are (matches between season)/(total unique whales in winter and summer). ΔYoY is the year-over-year rate of change in abundance, presented as 3-year moving average values for 2003–2020.

mid-sample year	unique winter whales	unique summer whales	matches between seasons	uncorrected abundance estimate	bias-correction factor (*F_t_*)	bias-corrected abundance estimate	ΔYoY	standard error bias-corrected abundance	between season match rate (%)
2002	1940	1252	175	13 820	1.22	16 875		5955	5.80
2003	3055	3103	526	18 001	1.06	19 065	6.9	2981	9.34
2004	4279	3236	757	18 279	1.06	19 340	7.6	3027	11.20
2005	5668	3236	913	20 078	1.05	21 063	3.4	843	11.43
2006	5391	2465	667	19 906	1.06	21 095	9.1	2660	9.28
2007	5052	2707	595	22 960	1.09	24 915	8.0	3081	8.31
2008	3769	2519	413	22 949	1.15	26 456	7.4	2976	7.03
2009	3268	2684	359	24 382	1.08	26 445	−2.5	4369	6.42
2010	2901	2754	324	24 601	0.94	23 007	4.2	4328	6.08
2011	3031	2605	284	27 725	1.07	29 636	8.9	4885	5.31
2012	3329	2541	277	30 450	1.10	33 488	10.1	4455	4.95
2013	3492	2466	294	29 212	1.09	31 701	1.7	6193	5.19
2014	4050	2494	359	28 077	1.11	31 243	−0.2	4835	5.80
2015	3877	2523	334	29 219	1.14	33 260	−1.9	5199	5.51
2016	4110	2607	392	27 282	1.09	29 852	−3.0	3290	6.20
2017	4125	2364	385	25 281	1.12	28 429	−7.1	6319	6.31
2018	5060	2512	549	23 125	1.16	26 731	−4.9	3342	7.82
2019	5087	2403	552	22 120	1.16	25 671	0.3	4427	7.96
2020	4989	2327	484	23 953	1.20	28 706	−0.1	4713	7.08
2021	4773	2640	567	22 198	1.20	26 662	−7.1	4192	8.28

### 3.2. Recapture rates and bias correction

Between-season matches ranged from 4.95% to 11.43% of individuals per season (table 3). Over the full study period, 63% of all individuals were captured in multiple seasons and/or regions, suggesting that a high proportion of the North Pacific humpback whale population was included in the sample [18]. While sample sizes were very limited for some regions, no region stood out with a disproportionately low recapture rate. Recapture rates ranged from 56.8% of individuals captured in multiple seasons and/or regions in the Mariana Islands to 87.2% in southern Mexico and Central America. Following the photo-ID methodology of [44], we assumed that 97–99% of potential matches were found.

Bias correction factors (*F_t_*) averaged 1.11 (0.94–1.22, s.d. 0.07, table 3), adjusting abundance estimations upward, in most cases, from uncorrected abundance estimates biased by non-representative sampling efforts.

### 3.3. Abundance estimation and trends

**Figure 2.**
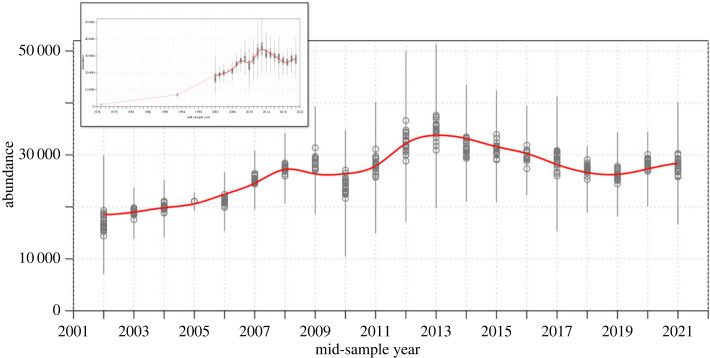
Bias-corrected estimates of humpback whale abundance for the full North Pacific Ocean. The red line represents a three-year moving average of abundance estimates. Vertical lines represent confidence intervals based on ± twice the standard errors (s.e.). S.e. include both the uncertainty in the abundance for the SPLASH years (CV = 0.04, mid-sample year = 2005 [30]) and the uncertainty in the bias-correction process. Inset: North Pacific humpback whale abundance in the context of post-whaling population estimates [25,26] and 1993 estimate [31].

**Figure 3.**
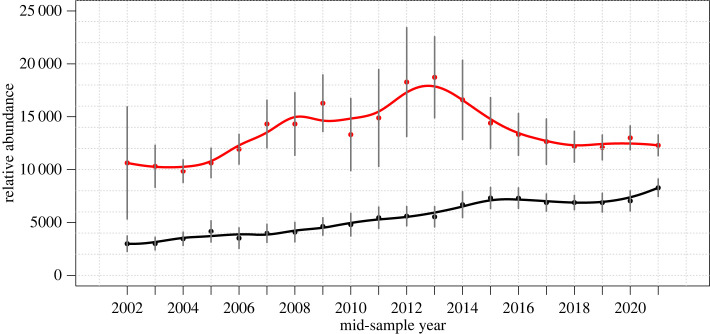
Relative estimates of humpback whale abundance for the Hawai‘i (red) and Mainland Mexico (black) regions based on mark–recapture comparisons with northern British Columbia and southeast Alaska, and California and Oregon, respectively. The lines represent 3-year moving averages of abundance estimates. Vertical lines represent confidence intervals based on ± twice the standard error (s.e.).

Bias-corrected estimates of humpback whales in the full North Pacific increased from 16 875 (s.e. = 5955) in sample year 2002 to a peak of 33 488 (s.e. = 4455) in 2012 before an inflection with a decline to 26 662 individuals (s.e. = 4192) by sample year 2021 (figure 2 and table 3). Growth from 2002 to 2013 was consistently positive apart from one anomalous year, 2010, averaging 5.9% yr^−1^. From 2014 to 2021, estimated abundance consistently declined, averaging −3.0% yr^−1^. Hawai‘i region relative abundance estimates showed a peak in 2013, then declined to abundance levels equal to the mid-2000s from 2017 onward (table 4 and figure 3). The Hawai‘i region growth phase was steeper than the North Pacific-wide aggregated dataset, averaging 6.9% yr^−1^ from 2002 to 2013, then declined at an average of −4.3% yr^−1^ from 2014 to 2021. By contrast, relative abundance for the Mainland Mexico region grew at an average of 7.1% yr^−1^ from 2002 to 2015 before appearing to stabilize with an average of 0.9% yr^−1^ growth from 2016 to 2021 (table 4 and figure 3).

**Table 4.** Estimates of relative abundance for the Hawai‘i and Mainland Mexico regions and the percentage of their total abundance represented by Hawai‘i. Values and standard errors (s.e.) are the same as in figure 3. ΔYoY is the year-over-year rate of change in estimated abundance, presented as 3-year moving average values for 2003–2020, with 2002 and 2021 omitted due to lack of multiple years to average creating excessive variabiliaty. Year over year growth rates above 11.8% exceed estimated maximum plausible population growth rates [52] and thus are expected to be estimation errors.

mid-year	Hawai‘i						Mainland Mexico						
	winter	summer	matches	abundance	s.e.	ΔYoY (%)	winter	summer	matches	abundance	s.e.	ΔYoY	% Hawai‘i
2002	1142	278	29	10 631	2650		491	570	93	2990	367		78
2003	1525	1431	211	10 309	996	−3.7	588	575	112	3003	298	7.8	77
2004	2123	1743	375	9853	534	0.0	736	604	128	3457	316	12.3	74
2005	2904	1290	352	10 625	697	5.3	906	521	113	4154	502	4.8	72
2006	3062	1097	281	11 927	706	13.8	887	371	93	3515	487	4.6	77
2007	2920	1028	209	14 314	1127	10.0	838	458	96	3971	430	−0.6	78
2008	2121	1132	167	14 312	1479	10.7	696	685	116	4088	459	9.6	78
2009	1591	1195	116	16 275	1338	−2.2	717	798	123	4627	411	6.5	78
2010	1080	1107	89	13 309	1703	1.3	733	731	111	4798	536	10.1	74
2011	1104	969	71	14 888	2291	4.5	811	764	113	5450	502	6.5	73
2012	1202	956	62	18 275	2572	11.7	1146	921	188	5596	453	4.6	77
2013	1364	1220	88	18 728	1914	3.3	1329	910	218	5534	478	7.4	77
2014	1922	1423	164	16 597	1872	−7.2	1368	975	199	6682	612	9.6	71
2015	1750	1454	176	14 395	1203	−10.9	1002	1632	223	7313	500	9.0	66
2016	1968	1339	197	13 327	986	−8.9	1052	2098	302	7296	484	1.0	65
2017	1985	1056	165	12 647	1070	−5.5	1169	2223	377	6885	404	−2.0	65
2018	2914	1635	391	12 167	730	−3.2	1421	2226	459	6885	337	−2.0	64
2019	3522	1784	519	12 094	588	1.0	1422	1889	390	6879	444	0.8	64
2020	4019	1606	496	12 999	566	0.3	1618	1869	429	7042	472	6.7	65
2021	4711	1593	610	12 294	492		1779	2283	490	8281	414		

### 3.4. Migration patterns and relative abundance

**Figure 4.**
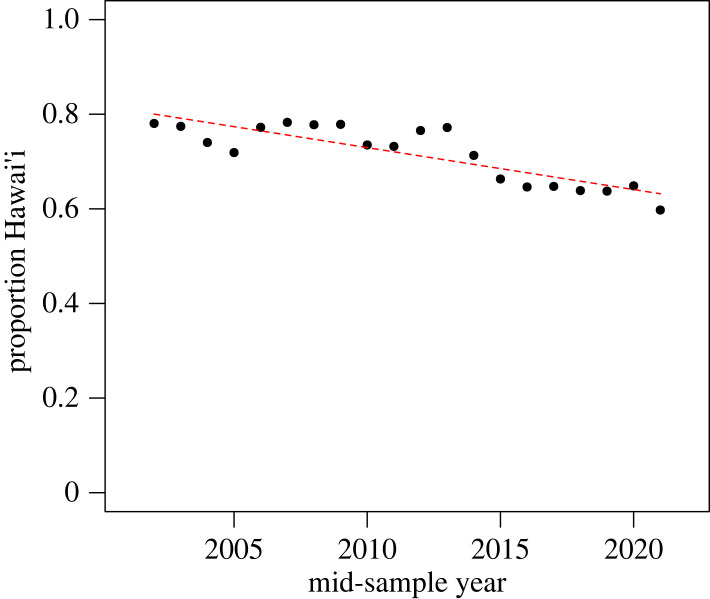
Proportion of Hawai‘i and Mainland Mexico relative abundance of humpback whales during study period of 3-year pooled samples 2002 through 2021, with a slope of −0.8% per year.

Analysis of migratory destinations of 2022 individuals found in southeast Alaska and northern British Columbia in 2004–2006 and 2025 individuals in the same area in 2019–2021 found 591 and 698 individuals in wintering areas, respectively, with no significant difference in migratory destinations between the two time periods (*χ*^2^ = 0.263, *p* = 0.877; table 5). The proportionate relative abundance of whales between Hawai‘i and Mexico in 2002–2006 was 78% versus 22%, respectively (table 4 and figure 4). The Hawai‘i percentage declined by an average of −0.8% annually over the study period, so that the relative abundances were 65% versus 35% respectively by the 2017–2021 sampling period (ANOVA *p* < 0.001).

**Table 5.** Migratory destinations of 2004–2006 and 2019–2021 southeast Alaska and northern British Columbia humpback whales show no change over the study period.

time period	SEAK/NBC total sample	total winter matches	Hawai‘i	Mainland Mexico	both Hawai‘i and Mainland Mexico
2004–2006	2022 individuals	29.2% (591)	90.2% (533)	10.3% (61)	0.5% (3)
2019–2021	2025 individuals	34.5% (698)	89.4% (624)	11.2% (78)	0.6% (4)

## 4. Discussion

This study estimated a total abundance of 26 662 (s.e. = 4192) humpback whales in the North Pacific as of 2021, growing from 2002 to 2021 at an average rate of 3% yr^−1^, with a 20% decline from a peak abundance of 33 488 (s.e. = 4455) in 2012. Across the North Pacific Ocean, we described a distinct inflection from post-whaling era rapid population recovery to a state where population dynamics may now be more constrained by environmental factors, including variability in resources induced by climate change. From 2002 through 2013, growth averaging 5.9% yr^−1^ agreed with the 5.2–8.6% yr^−1^ 95% confidence interval growth rate described by the SPLASH study [30] and was well within an estimated maximum biological potential growth rate of 11.8% yr^−1^ [52]. The timing of the inflection from long-term growth, most notably evident in our Hawai‘i relative population estimation (figure 3), is consistent with observed changes in oceanographic conditions in feeding areas, specifically the severe northeast Pacific Marine Heatwave (PMH) of 2014–2016 [53], which depressed biological productivity and thus prey availability in northeast Pacific Ocean whale feeding areas. Two other known sources of humpback whale mortality, ship strikes and entanglements, may have contributed to the apparent decline; both have exceeded estimated potential biological removal rates [54,55] and entanglement rates surged on the US West Coast in correlation with the PMH apparently due to habitat compression [43,56,57] but cannot account for an estimated population decline approaching 7000 individuals across the North Pacific in just 9 years (2012–2021).

The PMH, the most extreme in recorded history [41], had profound environmental impacts including elevated sea surface temperatures (by as much as 3 s.d. in some areas with maximum sea surface temperature anomalies sometimes exceeding 3–6°C to depths ranging from 50 to 200 m), a decline in sea surface winds, reduced upwelling and decreases in nutrient-rich water. This led to reduced phytoplankton biomass and restructured zooplankton communities in favour of lower-calorie species [58,59]. These changes, in concert with increased metabolically driven food demands of ectothermic forage fish resulting in quality and quantity declines in forage fish [60], and an increased demand for forage fish by large ectothermic predatory fish (e.g. groundfish and salmon), ultimately increased competition beyond the demands already imposed by top predators such as marine mammals and sea birds. The result created what Piatt *et al*. [61] proposed as an ‘ectothermic vise' on the diminished forage resource. High latitude waters within the PMH witnessed a decline in local abundance of humpback whales, where they traditionally feed on krill and small schooling fish such as herring, capelin, sand lance and juvenile salmon [39]. Reduced prey biomass of zooplankton combined with increased competition for diminished forage fish resulted in severely diminished presence of calves, prevalence of ‘skinny' whales, increased strandings [62], and the absence of individual whales with high site fidelity to specific locales. The impacts were also witnessed in humpback whale breeding grounds including significantly fewer whales and calves of the year (e.g. [38,63–65]). Co-occurring with the negative effects on humpback whales in their feeding grounds, several mass mortalities of fish-eating seabirds were documented suggesting far reaching impacts of the PMH. For example, over 60 000 dead or dying emaciated common murres (*Uria aalge*) were documented in 2015–2016 from California to Alaska, apparently from starvation, with high rates of reproductive failure [61]. The eastern Bearing Sea witnessed shifts in zooplankton community composition and forage fish distribution with a mass mortality of emaciated tufted puffins (*Fratercula cirrhata*) during the onset of molt, apparently associated with starvation [66]. Other species, whose food resources partially overlap with those diminished in association with the PMH, and which also experienced declines in body condition and unusual mortality rates included: various Alaskan large predatory groundfish, California sea lions (*Zaolphus californianus*) and Guadalupe fur seals (*Arctocephalus townsendi*) (summarized in [61]). Given all of these co-occurring impacts with the PMH, and because humpback whales feed on both krill and small schooling fish, and may be flexible in their ability to shift their dominant food resource between the two when faced with variations in oceanographic and ecological conditions affecting their food resources [67], they may serve as a valuable bellwether indicator species of ocean-basin ecosystem health. Their lack of recovery by 2021 is notable.

While conservation management has focused on populations defined by breeding areas [8], feeding area resource limitations are likely the greatest determinants of carrying capacity. Regional evidence in Alaska shows variable recovery in feeding areas where local abundance was severely reduced by the PMH, still depressed in Prince William Sound [40] compared to partial recovery in Glacier Bay National Park [39]. The documented downward inflection in the population growth curve of North Pacific humpback whales may indicate that population impacts of ecological stressors like the PMH may be broad, and supports evidence of persistence for years after oceanographic conditions return closer to the long-term mean [53]. Similar findings of the lingering effects of the PMH have been documented in seabirds in Cook Inlet, Alaska [68].

### 4.1. Bias correction validity and assumptions

The bias-correction approach allowed us to account for variable sampling effort over time and space, necessitating three assumptions. First, the assumption that the SPLASH abundance estimate is unbiased by non-representative geographical sampling is supported by an experimental design for the SPLASH study that pursued representative sampling of all wintering and summer areas known at the time [20,30]. The SPLASH study had no effort in the Mariana Islands, which has since been found to be a breeding area for humpback whales [21]; however, after seven seasons of effort there with 37 individuals identified [18], we are confident that this omission from SPLASH sampling effort would not significantly change the abundance estimation at the scale of the full North Pacific Ocean. The northwest Hawaiian Islands are now known to be a wintering area [69] with as yet unknown abundance and relationship to the main Hawaiian Islands. Of 37 individuals identified in two northwest Hawaiian Islands voyages of limited scope and duration (2007 and 2019), 24 individuals were resighted in Hawai‘i, interchange that suggests a substantial degree of mixing. We believe this is the only North Pacific humpback whale wintering area that may host an under-sampled, site-faithful population, though with a majority of identified individuals also encountered in the main Hawaiian Islands. Further study may thus reveal minimal impact on total abundance estimation statistics. Russian feeding areas were represented by only 102 individuals during SPLASH years [20]; in contrast, this study benefited from data from 2296 individuals [18]. This heterogeneity in sampling effort contributed to larger s.e. ranges derived from our jackknife approach in the full North Pacific abundance estimate, compared to narrower ranges in Hawai‘i and Mexico relative abundance estimates.

A further consideration of potential sampling bias is of equal detection probability. High accuracy of the AI image recognition tool used for matching [44] meant any fluking whale could be confidently identified irrespective of distinctiveness. Females with calves, however, are known to fluke less often in wintering areas [70] though Craig and Herman demonstrated that extended focal follows of non-fluking individuals would greatly reduce sampling bias [71]. The same study found lower wintering resight rates of females. Further, not all humpbacks migrate every year [72]. We believe sampling bias is effectively minimized by using multi-year pooled samples for each annual abundance estimation and the methodology of drawing from a dataset of nearly 200 000 identified encounters (192 869 unique encounters distilled to 132 684 unique season-year encounters) that may mimic the high detection rates of extended focal follows [71].

The second assumption, that migratory patterns have not changed, was conclusively found to be valid based on no difference between wintering destinations of over 2000 whales each in time periods near the beginning and end of our study period. This assumption is further supported by demonstrated strong maternal fidelity and natal philopatry in humpback whales with sufficient stability over time to show genetic differentiation [73–75].

Validity of the third assumption would require that population trends were uniform between and across all summer and wintering areas during the study period. We found that relative abundance between Hawai‘i and Mexico shifted from 78% versus 22% to 65% versus 35% over the course of the study (figure 4) apparently because of different population growth rates (figure 3). This 13% shift in relative abundance between Hawai‘i and Mexico, and possible undetected shifts in relative abundance between other study area regions, does violate this assumption and thus may have introduced error in our geographical bias correction factor affecting absolute abundance estimates. We realize that the 13% relative abundance shift is a violation of the assumption of consistent geographical distribution underlying our sampling bias correction methodology; however, we believe its effects on observed abundance trends (as documented by the study) are likely to be minimal because (i) the large samples created by multi-year pooled effort where approximately one-third to half of the total estimated North Pacific humpback population was captured in most 3-year samples and (ii) the bias corrections themselves are relatively small. The overall trends without the bias correction are similar to the bias-corrected trends (table 2), although the values are lower in all years except one. We cannot fully discount this as a potential source of bias, particularly after 2013 when the proportion of the population in Hawai‘i decreased substantially (figure 4). To address these sources of uncertainty, future study for humpback whales in the North Pacific should include absolute abundance estimation by region.

### 4.2. Relative abundance estimation

We sought to refine the ocean-basin-wide abundance estimate with regional estimates based on the two best-sampled subset wintering areas, Hawai‘i and the Mainland Mexico. These subset models could not be considered absolute abundance estimations because each 3-year sample was not assumed to be a closed population or controlled for sample bias such as, in the case of Mainland Mexico, an overlap of Central American whales. These whales are known to co-occur using the region as a migratory corridor [46,76], and therefore the subset abundance estimate sampled a population with undefined boundaries. In Hawai‘i, sample effort was concentrated in the Maui-Nui channel and to lesser extents off the islands of Hawai‘i, Oahu and Kauai, without a clear definition of whether this is a comprehensive sample of an equally mixed Hawaiian island population. The value of the relative abundance estimations was in describing different population growth trajectories with far lower s.e. estimations than the North Pacific-wide model. An apparent sharp decline in humpback whale relative abundance off west Maui (which traditionally has hosted the greatest concentrations of humpback whales including calves of the year, e.g. [77–79]) was reported after the 2014–2016 PMH, using boat-based transect surveys [38] and passive acoustic monitoring [65], as well as off the Kohala coast of Hawai‘i Island using shore-based systematic scans [64]. The relative abundance trend measured agrees with data from density estimates by fixed-wing aerial surveys of the entire Maui Nui region for 1993–2003 and 2019–2020 [80], though these surveys did not occur during the period of apparent steep increase then decrease, over the 2005–2016 period. Our results support the conclusions of Frankel *et al*. [64] that environmental variables likely caused Hawaiian humpback populations to decline due to the PMH. Fluctuating but resilient humpback whale crude birth rates in Hawai‘i [38,80] suggest the measured −4.3% yr^−1^ decline from 2014 to 2021 may be temporary, stabilizing if ocean conditions in feeding areas return to the long-term mean. These trends point to an apparent ecological shift from a half century of recovery response following near-extirpation by industrial whaling to carrying capacity-limited abundance. It is notable that the end of the shift did not appear to occur from population growth but rather from a decrease in carrying capacity triggered by rapid climate change.

## 5. Conclusion

The end of the industrial whaling era left oceans largely empty of great whales [23,24]. Yet with no species hunted to global extinction during this period, full recovery became a possibility. For humpback whales, steady population growth was the 40-year trend in the North Pacific, and it is to be celebrated that humpback whales appeared sufficiently recovered to qualify for partial delisting from legal protected status regimes in the USA and Canada. Ironically, the timing of legal conservation status changes for the species was coincident with the dramatic ocean-warming-induced decline documented in this study. Some populations of humpback whales may no longer be a priority for endangered species conservation funding [81], but now offer high value as an indicator species of ocean-basin-wide ecosystem health [82] in a world where we can expect increased frequency and severity of marine heatwaves. Past population monitoring efforts offered periodic abundance estimates interspersed with many years with very coarse assessment of population trajectory (figure 2). This study creates a shift to continuous population monitoring with rapid feedback, enabled by an advance in AI-assisted photo-ID [44] and an ocean basin-wide collaborative effort [18]. If this cost-effective collaborative effort is maintained with funding to gather robust sample sizes from most wintering and summer areas of the species, we can expect to be able to detect shifts in abundance in near real time through ongoing updates of the model with data from each passing year.

This study establishes a beginning, and represents just one of many possible applications of a rich and continuous collaborative dataset to monitor humpback whale abundance. Aggregation of historical data together with a coordinated future study could enable regional abundance estimations, fine-scale understanding of migratory patterns, and time-varying survivorship and mortality rates. Our method of estimating population size was deliberately based on the simplest possible approach (pooling all wintering and summer areas) in order to be comparable to the most recent previous estimate of humpback whale abundance for the North Pacific [30]. There were substantial limitations to this approach: it did not provide region-specific estimates of abundance or trends, it was a closed-population model and did not allow for estimation of mortality or birth rates, and individual estimates for a given year did not use information available in the longer time-series to improve the estimate for that year. It is our hope and expectation that this is just the first of many explorations of this rich and living dataset. Continued collaborative efforts across North Pacific wintering and feeding areas have great potential for population monitoring and as an indicator of ecosystem health; some of this is already underway. The In-depth Assessment process of the International Whaling Commission Scientific Committee is currently developing a population model seeking to include a changing carrying capacity term that accounts for environmental variability [29]. And a current initiative in Hawai‘i to fit an integrated population model to these data may refine understanding of regional abundance in the context of post-delisting monitoring under the ESA [83].

## Data Availability

Data and code are accessible here: https://github.com/tedcheese/RSOS-NPAC-abundance. Supplementary material is available online [84].
